# Growth and metastasis of B16-F10 melanoma cells is not critically dependent on host CD73 expression in mice

**DOI:** 10.1186/1471-2407-14-898

**Published:** 2014-12-02

**Authors:** Sandra Burghoff, Xuan Gong, Claudia Viethen, Christoph Jacoby, Ulrich Flögel, Sabine Bongardt, Anne Schorr, Andreas Hippe, Bernhard Homey, Jürgen Schrader

**Affiliations:** Institute of Molecular Cardiology, Heinrich Heine University Duesseldorf, 40225 Duesseldorf, Germany; Department of Dermatology, University Hospital Duesseldorf, 40225 Duesseldorf, Germany; MVZ Labor Dessau GmbH, 06847 Dessau, Germany

**Keywords:** CD73, B16-F10 melanoma, Adenosine, Immune system, Tumor, Mice

## Abstract

**Background:**

Recent studies have suggested that adenosine generated by ecto-5′-nucleotidase (CD73) in the tumor microenvironment plays a major role in promoting tumor growth by suppressing the immune response and stimulating angiogenesis via A2A and A2B receptors. However, adenosine has also been reported to inhibit tumor growth acting via A1 and A3 receptors. Therefore the aim of this study was to clarify the role of host CD73, which catalyzes the extracellular hydrolysis of AMP to adenosine, on tumor growth and metastasis of B16-F10 melanoma cells.

**Methods:**

CD73 and alkaline phosphatase (AP) activity of B16-F10 melanoma cells were measured by HPLC. Tumor cells were injected either subcutaneously or intradermally in WT and CD73^−/−^ mice and tumor growth was monitored by MRI at 9.4 T. Immune cell subpopulations within tumors were assessed by FACS after enzymatic digestion. An endothelium specific CD73^−/−^ was created using Tie2-Cre^+^ mice and CD73^flox/flox^ (loxP) mice. Chimeric mice lacking CD73^−/−^ on hematopoietic cells was generated by bone marrow transplantation. Lung metastatic spread was measured after intravenous B16-F10 application.

**Results:**

B16-F10 cells showed very little CD73 and negligible AP activity. Neither complete loss of host CD73 nor specific knockout of CD73 on endothelial cells or hematopoietic cells affected tumor growth after subcutaneous or intradermal tumor cell application. Only peritumoral edema formation was significantly attenuated in global CD73^−/−^ mice in the intradermal model. Immune cell composition revealed no differences in the different transgenic mice models. Also lung metastasis after intravenous B16-F10 injection was not altered in CD73^−/−^ mice.

**Conclusions:**

CD73 expression on host cells, particularly on endothelial and hematopoietic cells, does not modulate tumor growth and metastatic spread of B16-F10 melanoma cells most likely because of insufficient adenosine formation by the tumor itself.

**Electronic supplementary material:**

The online version of this article (doi:10.1186/1471-2407-14-898) contains supplementary material, which is available to authorized users.

## Background

Cancer cells are able to promote their own survival by changing the tumor microenvironment to their own favor. One important autocrine and paracrine factor is the nucleoside adenosine, which accumulates in significant quantities in the tumor environment together with its precursor ATP to finally act on P1 (adenosine) and/or P2 (ATP) receptors [1]. In recent years it became increasingly evident that ATP is released from viable tumor cells [2] but also from activated immune cells [3] and subsequently becomes degraded to adenosine by the ecto-nucleotidases CD39 and CD73 expressed on tumor and immune cells [4]. Locally formed adenosine is assumed to suppress antitumor immune response, to promote angiogenesis thereby inhibiting immune-mediated damage which ultimately promotes tumor progression [5]. In favor of this hypothesis is the finding that immunogenic melanoma cells when subcutaneously inoculated showed retarded tumor growth and increased survival in A2A^−/−^ mice [6]. A2B^−/−^ mice also showed attenuated tumor growth and vascularization when lung carcinoma cells were subcutaneously applied [7]. On the other hand, adenosine reduced glioblastoma growth most likely acting via the A1 receptor in microglia [8]. Furthermore, treatment with an A3 agonist significantly reduced growth of melanoma cells in immune-competent wildtype (WT) mice [9]. Therefore the outcome of an adenosinergic microenvironment will critically depend on the expression of adenosine receptor subtypes on the various immune cell subpopulations and most likely also on the immunogenicity of tumor cells.

Lately, interest has been shifted towards the sources of extracellular adenosine, especially the ecto-5′-nucleotidase (CD73). This ectoenzyme catalyzes dephosphorylation of extracellular AMP to adenosine, thereby controlling the decisive step in the extracellular degradation of ATP to adenosine [5]. The role of CD73 expression on tumor cells has been studied by several groups and its stimulating influence on tumor growth and metastasis was demonstrated in vitro and in vivo [10–12]. Recent studies have investigated the role of CD73 expressed on host cells in different tumor cell models using CD73^−/−^ mice [13–16], pharmacologic inhibition of CD73 [17] or anti-CD73 antibodies [17], which all suggest a stimulating effect of host CD73 on local tumor growth and metastatic spread. In particular, tumor cells (colon adenocarcinoma, melanoma, lymphoma) which additionally express immunogenic antigens showed an impressive positive effect of host CD73 on tumor growth, especially when compared to their respective less-immunogenic parent tumor cell lines [14, 15]. Yet, not all studies showed a clear promoting role of CD73 on tumor progression, especially when using less immunogenic tumor cell lines [14, 15]. It also should be noted that systemic application of inhibitors or antibodies targets CD73 on tumor cells as well as host immune cells and vascular endothelium to the same extent. Finally, clinical studies tested CD73 expression on tumor cells as a prognostic marker has yielded contradictory results [18, 19]. In view of these findings, there is clearly a need to further specify the role of host CD73 on local tumor growth and metastatic spread.

The aim of the current study was to investigate the role of host CD73 on tumor progression of B16-F10 melanoma cells, a well-established tumor model for melanoma growth in C57BL/6 mice [20, 21]. To address this question, global and endothelium-specific CD73^−/−^ mutant mice together with bone marrow chimeras to selectively delete CD73 on hematopoietic cells were used. For the precise assessment of tumor size and peritumoral edema ^1^H magnetic resonance imaging (MRI) at 9.4 T was used. Because the orthotopic site of spontaneous malignant melanoma is the dermoepidermal junction zone, we also injected tumor cells intradermally, thereby providing a condition which may better mimic spontaneous tumor progression of melanoma. To investigate hematogenous metastasis we also measured pulmonary seeding after intravenous injection of melanoma cells.

## Methods

### Animals

All experiments were performed with approval of the local government committee, the “Landesamt fuer Natur, Umwelt und Verbraucherschutz Nordrhein-Westfalen”.

Generation of C57BL/6 J CD73^−/−^ mice has been described previously [22]. For generation of endothelium specific CD73^−/−^ (eCD73^−/−^) mice, Tie2-Cre^+^ mice [23] were crossed with CD73^flox/flox^ (loxP) mice (controls) [22] to generate Tie2-Cre^+^ CD73^flox/WT^ mice. Tie2-Cre^+^ CD73^flox/WT^ mice were then crossed with CD73^flox/flox^ mice to generate Tie2-Cre^+^ CD73 ^flox/flox^ (eCD73^−/−^) mice which specifically lack CD73 only on endothelial cells. Targeted deletion of CD73 on endothelial cells of eCD73^−/−^ mice was confirmed by immunohistochemistry (Additional file 1: Figure S1).

### Cell culture

Murine B16-F10 melanoma cells (ATCC, USA) were cultured in DMEM/10% (v/v) fetal calf serum/2 mM L-glutamine/100 U/ml penicillin/100 μg/ml streptomycin (all Life Technologies, Germany) at 37°C and 5% (v/v) CO_2_.

### Enzyme activity assays

B16-F10 cells (5 × 10^4^) were incubated in RPMI-1640/10% (v/v) fetal calf serum/2 mM L-glutamine for 24 h at 37°C and 5% (v/v) CO_2_. Thereafter, medium was changed and cells were incubated for another 20 min. To inhibit AMPase activity 50 μM levamisole or 50 μM AOPCP was used. The reaction was started with 50 μM etheno-AMP. After 10 min, 20 min, 30 min, 40 min and 50 min 15 μl supernatant was removed, immediately mixed with the same volume 1 M perchloric acid and stored at −20°C until further analysis.

Samples were neutralized with 1 M K_3_PO_4_ prior to HPLC analysis. Etheno-AMP and etheno-adenosine amounts were determined using a 1525 Binary HPLC pump (Waters, Germany) which was connected to Waters 2475 multi wavelength fluorescence detector (λ_Em_ = 410 nm, λ_Ex_ = 280 nm). Separation of the two analytes was achieved on an XTerra MSC18 Column (Waters, 5 μm, 4.5 × 100 mm). Solution A contained 6% (v/v) acetonitrile/5.7 mM tetrabutylammonium bisulfate/30.5 mM KH_2_PO_4_, pH 5.8. Solution B consisted of 66% (v/v) acetonitrile/5.7 mM tetrabutylammonium bisulfate/30.5 mM KH_2_PO_4_, pH 5.8. At a flow rate of 0.75 ml/min separation was achieved by a linear gradient from 0 to 34% (v/v) solution B for 5.6 min and 34% (v/v) solution B for 1.4 min. Areas from the appropriate chromatograms were used for determination of reaction rate which is given as enzymatic activity per 1 × 10^6^ cells by using Waters Breeze software. Each sample was measured in triplicate.

### Bone marrow transplantation

Bone marrow cells from WT and CD73^−/−^ mice were isolated from shinbone and calf bone of female donor animals, washed in PBS and filtrated through a 70 μm nylon mesh. 1 × 10^7^ cells were intravenously injected (tail vein) into male host animals, which were irradiated (8 Gy) 24 h before. For infection prophylaxis 80 mg/l vancomycin (Hexal, Germany) and 40 mg/l enrofloxacin (Bayer, Germany) were given with drinking water starting one week before irradiation and lasting five weeks post bone marrow transplantation. Success of transplantation was verified by FISH analysis using peripheral blood after the individual experiments. All male animals were 14 weeks old for tumor cell injection and had > 97% of female blood cells demonstrating the success of the transplantation.

### Tumor models

Appropriate numbers of B16-F10 cells were suspended in PBS and checked for viability using trypan blue staining. Only when cell viability was > 90% the cell batch was considered for injection. For subcutaneous or intradermal application skin of mice at an age of 6–10 weeks was shaved at the site of injection. Three different tumor models were used: (1) for subcutaneous injection 25 × 10^4^ cells in 100 μl PBS were injected into the right hind limb right beneath the hip, (2) for intradermal injections 1 × 10^6^ cells in 100 μl PBS were injected into the right flank, (3) for intravenous injection 25 × 10^4^ cells in 250 μl PBS were injected into the tail vein of the animals. In the first two models growth of the tumor was assessed over time by MRI. At the end of the experiments, tumors were isolated and used for the analysis of immune cell subpopulations by FACS.

### Tumor volume determination using MRI

For determination of tumor volume and tumor edema volume ^1^H MRI was performed and images were planimetrically analyzed. Mice were anesthetized with 1.5% (v/v) isoflurane in a water-saturated gas mixture of 30% (v/v) oxygen in nitrogen applied at a rate of 75 ml/min by manually restraining the animal and placing its head in an in-house-built nose cone. Measurements were performed in a vertical Bruker DRX 9.4 T wide-bore NMR spectrometer which was equipped with a 40 mm gradient system and a linear 30 mm saw-resonator. For 2D-multislice-RARE sequences (identical to the turbo-spin-echo-sequence) the following parameters were used: repetition time (TR) = 3.5 s, echo time (TE) = 15.8 ms, field of view (FOV) = 3 × 3 cm^2^, matrix size = 256 × 192 (zero filling to 256 × 256 resulted in a pixel size of 117 × 117 μm^2^), acquisition time = 2.8 min. The tumor was scanned in 30 consecutive axial 1-mm slices. For evaluation of volumes areas of solid tumor and edema tissue were determined in each slice and volume was calculated taking the slice thickness of 1 mm into account.

### Tumor cell separation, FACS analysis and IFN-γ-ELISpot

After cervical dislocation of mice, tumors were excised, minced and then incubated in an enzyme mixture (7.5 mg collagenase type I, 2.5 mg hyaluronidase type I-S (both Sigma-Aldrich, Germany) in 10 ml PBS) at 37°C for 1 h. After filtration of the cell suspension through a 100 μm nylon mesh erythrocytes were lyzed in 155 mM NH_4_Cl, 10 mM KHCO_3_, 0.1 mM Na_2_-EDTA (pH 7.4, 4°C, 8 min) and washed with PBS.

For FACS analysis cells were stained using the following anti-mouse antibodies: anti-CD4-FITC, CD8a-FITC, CD11b-FITC, CD11c-FITC, CD45-APC (all BD, Germany), CD25-PE (Miltenyi Biotec, Germany), as well as appropriate isotype controls. Cells were fixed in 4% (v/v) paraformaldehyde after staining. Analysis was performed on a BD FACSCanto II using the FACS Diva Software (both BD, Germany). Gates of immune cell subpopulations were defined by staining with respective isotype control antibodies and fractions are given as percentage of CD45^+^ immune cells.

For the IFN-γ-ELISpot 2 × 10^5^ cells of the tumor were used and protocol was run according to the manufacturer’s specification (R&D Systems, Germany). The number of IFN-γ secreting cells was determined by counting the number of visible spots on the plate.

### Statistics

All results are presented as mean values ± standard error of the mean (SEM). Results were analyzed by two-tailed Student’s t-test. Differences were considered to be statistically significant at a value of p < 0.05.

## Results

### B16-F10 cells have low intrinsic AMPase activity

**Figure 1:**
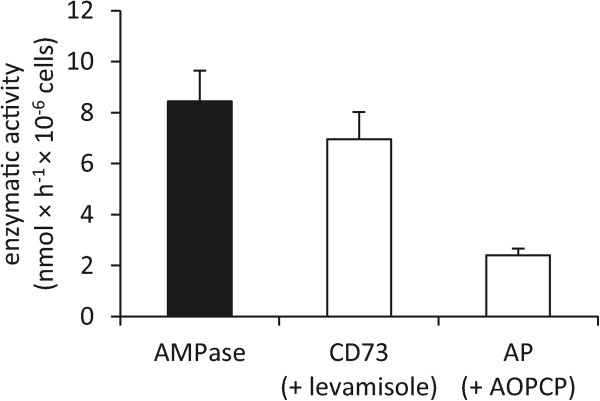
**Low ecto-AMPase activity in B16-F10 cells.** AMPase activity was measured by the dephosphorylation of etheno-AMP to etheno-adenosine in cultured B16-F10 cells using HPLC. CD73 and alkaline phosphatase (AP) activity were assessed using the AP inhibitor levamisole (50 μM) and the CD73 inhibitor AOPCP (50 μM) respectively. Data are shown as mean ± SEM (n = 3).

For analysis of intrinsic ecto-AMPase activity in B16-F10 tumor cells, enzymatic conversion of etheno-AMP to etheno-adenosine was measured by HPLC. As shown in Figure 1, AMPase activity (8.4 ± 2.1 nmol × h^−1^× 10^−6^ cells) on B16-F10 cells was rather small, particularly when comparing it with the extracellular ATP concentrations reported to be within the hundreds of micromolar range in solid tumors [24]. CD73 activity was measured by blocking alkaline phosphatase (AP) with levamisole and AP activity was determined by using the specific CD73 inhibitor AOPCP. B16-F10 cells catalyzed dephosphorylation of AMP mainly by CD73 (7.0 ± 1.8 nmol × h^−1^× 10^−6^ cells) while their AP activity was negligible. Our finding of a rather low CD73 activity is in accordance with results from others who failed to detect any CD73 expression on B16-F10 cells by less sensitive techniques [14, 16].

### Role of host CD73 on tumor growth after subcutaneous injection of B16-F10 cells

**Figure 2:**
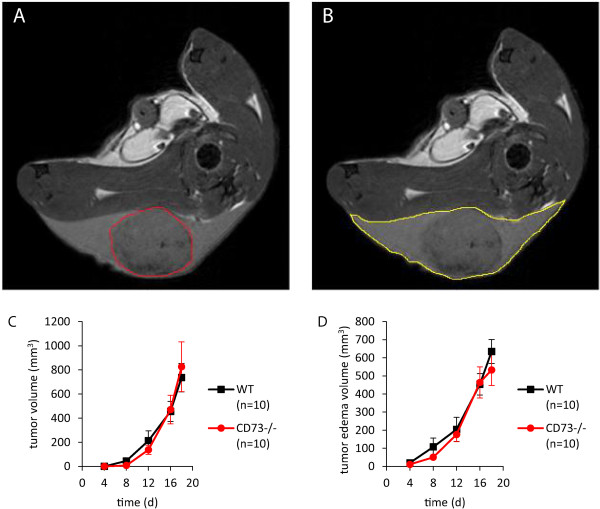
**Global loss of host CD73 did not alter subcutaneous B16-F10 tumor growth.** B16-F10 cells (25 × 10^4^) were injected subcutaneously into the hindlimb of WT and CD73^−/−^ mice. **(A, B)** Representative MRI measurements of the tumor and peritumoral edema were performed for 18 days post injection. Tumor volume and peritumoral edema are encircled in red and yellow respectively **(C, D)**. No differences in tumor volume or peritumoral edema volume were observed (n = 10).

**Figure 3:**
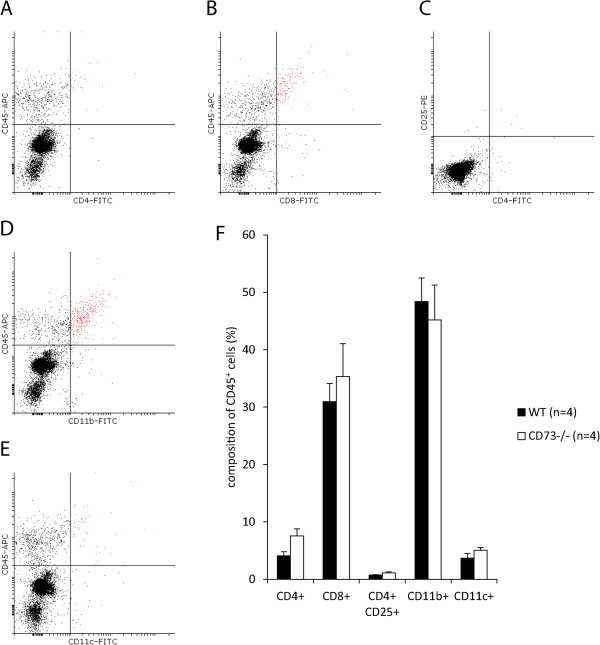
**Loss of host CD73 did not alter intratumoral immune response of B16-F10 tumors.** Subcutaneously injected B16-F10 tumors, reported on in Figure 2, were excised on day 18 post injection, minced and enzymatically separated into single cell suspension. Immune cell subsets were measured with FACS. Representative dot plots of CD4^+^ T helper **(A)**, CD8^+^ cytotoxic T cells **(B)**, CD4^+^ CD25^+^ T cells **(C)**, CD11b^+^ **(D)** and CD11c^+^ myeloid cells **(E)** are shown (gated cells are marked in red). **(F)** No differences in immune cell composition of tumors from WT and CD73^−/−^ mice were obtained. Data are shown as mean ± SEM (n = 4).

Because B16-F10 cells exhibit only minimal CD73 activity, this enabled us to study the role of host CD73 on tumor growth. To this end B16-F10 cells were injected subcutaneously into the hindlimb of WT and CD73^−/−^ mice at a concentration (25 × 10^4^ cells) previously used by others [9, 14, 17, 25]. Tumor growth and peritumoral edema formation was monitored by MRI at 9.4 T for 18 days, as shown in representative images in Figure 2A-B. From the quantitative data summarized in Figure 2C-D it is obvious that there were no differences between WT and global CD73^−/−^ mice when either tumor volume (Figure 2C) or peritumoral edema formation around the solid tumor was measured (Figure 2D).At the end of the experiments, B16-F10 tumors were excised and immune cell distribution within the tumor was measured by FACS (Figure 3A-E). As shown in Figure 3F, we found no differences between the two experimental groups regarding the distribution of lymphoid and myeloid immune cell subsets, thus suggesting no changes of immune response in absence of host CD73.

**Figure 4:**
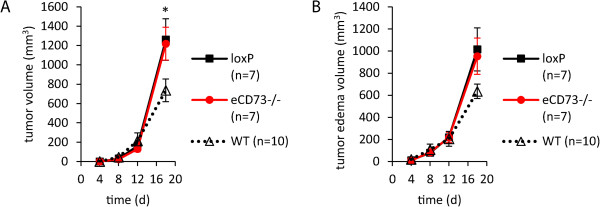
**Loss of CD73 on vascular endothelial cells did not affect B16-F10 tumor growth. (A, B)** B16-F10 cells (25 × 10^4^) were injected subcutaneously into the hindlimb of loxP and endothelium specific CD73^−/−^ (eCD73^−/−^) mice and MRI measurement of tumor and peritumoral edema were performed for 18 days post injection (n = 7). Compared with WT mice (n = 10) loxP and eCD73^−/−^ mice showed a significantly increased tumor growth (p = 0.04 for loxP and p = 0.03 for eCD73^−/−^ mice respectively on day 18) and a trend towards an increased peritumoral edema formation (p = 0.07 and p = 0.08 respectively on day 18). Data are shown as mean ± SEM. *p < 0.05.

Similar experiments as with the global CD73 knockout were carried out in mice in which CD73 on vascular endothelium was selectively deleted (Tie2-Cre^+^ CD73 ^flox/flox^ mice). As shown in Figure 4A-B, there were no differences in tumor growth and peritumoral edema formation between the loxP control and the endothelium specific knockout mice. Note, however, that tumor growth and edema formation in both experimental groups were higher at day 18 when compared with respective values in WT controls (Figure 4A-B), suggesting an influence of the genetic background.

**Figure 5:**
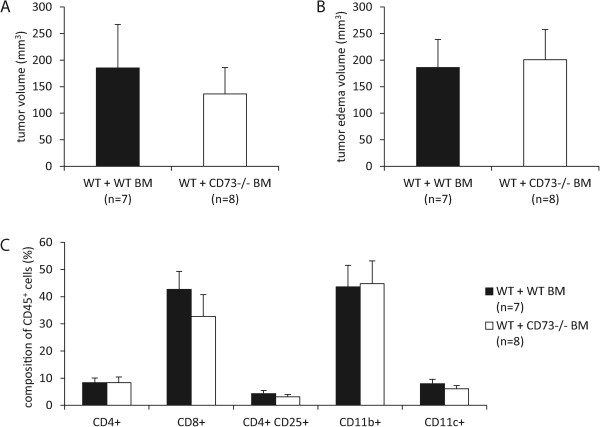
**Loss of CD73 on hematopoietic cells did not affect B16-F10 tumor growth and immune response.** WT or CD73^−/−^ bone marrow was grafted to WT mice after whole-body irradiation. B16-F10 cells (25 × 10^4^) were injected subcutaneously into the hindlimb of bone barrow grafted mice. **(A, B)** MRI measurement of tumor and peritumoral edema was performed on day 17 post injection. **(C)** No differences in immune cell composition within tumor were observed in FACS. Data are shown as mean ± SEM (n = 7 – 8).

That the genetic background can influence the growth of B16-F10 cells became also obvious when we used chimeric mice in which bone marrow from CD73^−/−^ mice was transplanted to WT mice, resulting in the loss of CD73 on hematopoietic cells. As appropriate controls, WT mice were treated accordingly and received autologous bone marrow from the WT genotype. In all cases, bone marrow from female mice was transplanted into male mice and the effectiveness of the transplantation was controlled by FISH. Using the identical protocol of B16-F10 cell inoculation as above, we again found no significant differences between the two experimental groups after 17 days in tumor volume, edema formation and infiltrating immune cells (Figure 5A-C). However, tumor growth and edema formation in WT mice transplanted with WT bone marrow was significantly reduced when compared with the controls in respective experiments in global (Figure 2C-D) and endothelium specific CD73 mutants (Figure 4A-B).

### Intradermal injection of B16-F10 cells influences tumor edema but not tumor growth

**Figure 6:**
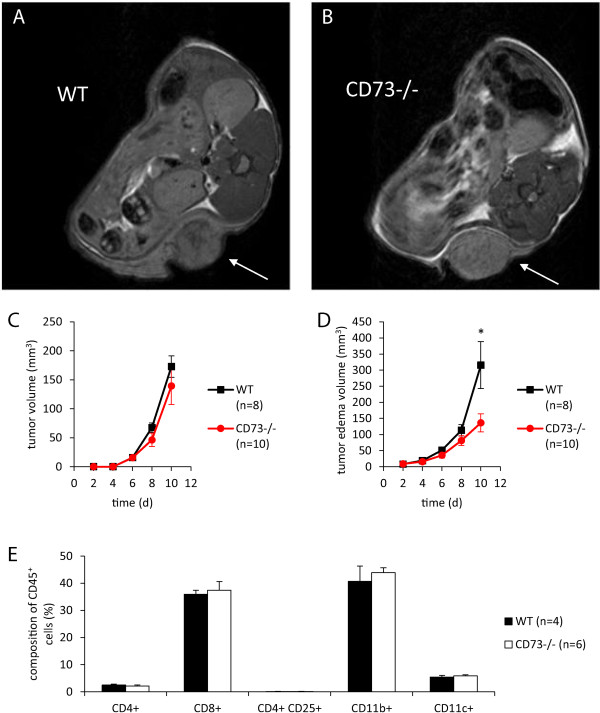
**B16-F10 peritumoral edema but not tumor volume was reduced in CD73** ^**−/−**^ **mice after intradermal injection.** B16-F10 cells (1 × 10^6^) were injected intradermally into the flank of WT and CD73^−/−^ mice. MRI measurement of tumor and peritumoral edema was performed for 10 days post injection. Representative MR images of one WT **(A)** and one CD73^−/−^ **(B)** mouse bearing tumor (arrow) are shown. **(C, D)** Tumor volume was not altered by lack of CD73 in mice, while peritumoral edema was significantly decreased in CD73^−/−^ mice (n = 8 – 10). **(E)** No differences were observed in immune cell subsets analyzed in tumors from WT and CD73^−/−^ mice (n = 4 – 6). Data are shown as mean ± SEM. *p < 0.05.

Using a potentially more immunogenic approach, we next inoculated B16-F10 cells intradermally into the flank of mice (Figure 6A-B) as previously described [26]. Although absence of host CD73 did not affect tumor growth (Figure 6C), peritumoral edema was found to be significantly attenuated in CD73^−/−^ mice (Figure 6D). Similar to the subcutaneous approach, we observed no differences in tumor immune cell response between WT and CD73^−/−^ mice (Figure 6E).

**Figure 7:**
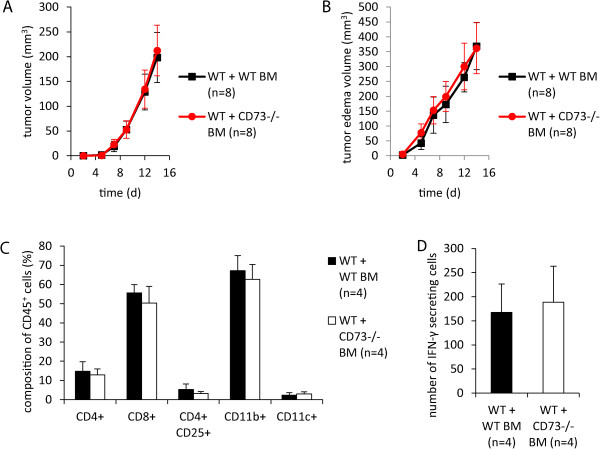
**Loss of CD73 on hematopoietic cells did not reduce peritumoral edema after intradermal B16-F10 application.** WT mice received bone marrow graft from WT or CD73^−/−^ mice after whole-body irradiation. B16-F10 cells (1 × 10^6^) were injected intradermally into the flank of bone barrow grafted mice. MRI measurement of tumor and peritumoral edema was performed over 14 days post injection. **(A, B)** Tumor and peritumoral edema volume, respectively (n = 8). **(C)** Loss of CD73 on hematopoietic cells did not alter intratumoral lymphoid and myeloid immune cell subsets (n = 4). **(D)** IFN-γ-ELISpot was performed in single cell suspensions of digested tumors containing both tumor and infiltrating immune cells. No difference was observed in the number of IFN-γ secreting cells in both groups (n = 4). Data are shown as mean ± SEM.

We next investigated whether the observed effect on tumor edema was caused by the loss of CD73 on hematopoietic cells. Therefore bone marrow transplanted chimeric mice (see above) were challenged with intradermally applied B16-F10 tumor cells. As expected, tumor growth did not differ in both groups (Figure 7A). However, no difference in peritumoral edema was observed in mice reconstituted with CD73^−/−^ bone marrow (Figure 7B). Thus, CD73 on non-hematopoietic cells, most likely endothelial cells, mediated the observed effect on peritumoral edema (Figure 6D). Differences in intratumoral immune response cannot explain this observation, since FACS analysis and IFN-γ-ELISpot of tumors from both experimental groups showed no differences (Figure 7C-D).

### Loss of host CD73 did not affect lung metastasis of B16-F10 cells

**Figure 8:**
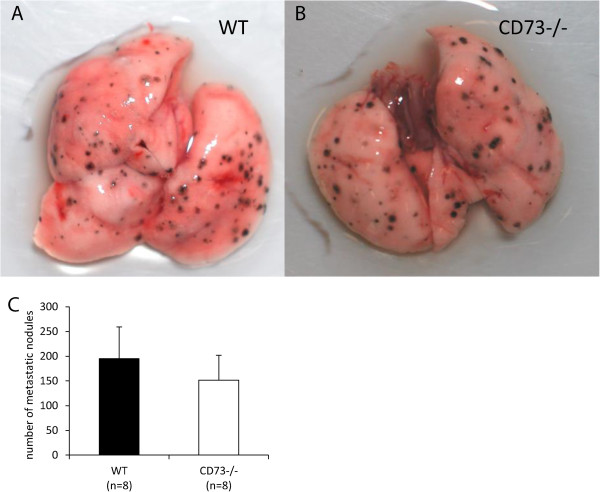
**Loss of host CD73 did not alter lung metastasis.** 25 × 10^4^ B16-F10 cells were injected into the tail vein of WT and CD73^−/−^ mice. Lungs of mice were removed after 10 days and the number of visible black metastatic nodules on the lung surface was counted. Representative images of lungs from one WT **(A)** and one CD73^−/−^ **(B)** mouse is shown. **(C)** No differences in the metastatic load of lungs were observed between WT und CD73^−/−^ mice. Data are shown as mean ± SEM (n = 8).

Finally we investigated the role of CD73 on hematogenic metastasis by injecting 25 × 10^4^ B16-F10 cells intravenously into mice and harvesting the lungs after 10 days. As shown in the representative Figure 8A-B and the statistical evaluation in Figure 8C, no difference was observed in the number of metastatic nodules on the lung surface between WT and CD73^−/−^ mice.

## Discussion

Experimental evidence summarized in several recent reviews [4, 27, 28] suggests that inhibition of CD73, which catalyzes the extracellular production of adenosine from AMP, has the potential to reduce tumorigenesis and metastasis by enhancing antitumor T cell immunity via adenosine receptor signaling. This hypothesis is supported by studies in mouse models using melanoma (B16-F10) in mutants lacking CD73 [13–16] or inhibitors of CD73 [17] or systemically applying anti-CD73 antibodies [17]. On the other hand, B16-F10 cells which were altered to overexpress CD73 showed no differences in local tumor growth [10]. The present study reports, that specific loss of host CD73 in different transgenic mouse models has no significant influence on tumorigenesis when B16-F10 melanoma cells were injected either subcutaneously or intradermally or intravenously.

Similarly to data in the literature [14, 16, 17] we injected 25 × 10^4^ B16-F10 melanoma cells subcutaneously but found no differences in tumor growth and immune cell subset composition within the tumor when using a global CD73 knockout, an endothelium specific CD73 knockout or a CD73 knockout on hematopoietic cells generated by bone marrow transplantation. In particular, no differences were observed in the important cell fractions involved in antitumoral immune response: CD4^+^ T helper, CD8^+^ cytotoxic T and CD4^+^ CD25^+^ T cells. Whether there were changes in distinct polarized macrophage subsets has not been investigated by us. Overall our data suggest that neither the CD73 on invading immune cells nor the CD73 on tumor vessels influenced local growth of the melanoma cells. Consistent with this finding we found that intradermal injection of B16-F10 melanoma cells did not alter tumor growth in the CD73 mutant.

The reasons for our surprising findings are not immediately obvious. In the present study we took great care to standardize the experimental procedures by using only freshly thawed B16-F10 cells (obtained from ATCC) of the same passage, which were regularly checked for viability and mycoplasma. In the different experimental series, melanoma cells were kept at 4°C and were injected within 2 hours after cell collection to minimize changes in cell quality. We also purposely alternated the injection between WT and knockout mice on a one by one basis to achieve a homogenous distribution between the experimental groups. Finally we have measured tumor expansion in a sufficient number of animals (n = 10) with MRI which permitted the precise volume determination over time when compared with caliper measurement in literature [13–17]. Whether differences in cell handling and experimental protocol may explain some of the differences reported in the literature [13–17] cannot be decided on the basis of published data.

Aside from differences in the experimental protocol additional factors may have also played a role. It is well known that the genetic background can influence tumor growth [29, 30]. The CD73 mutants used in the published melanoma studies were generated by different gene deletion strategies [22, 31], so that positional effects might have influenced the outcome. In the present study we have observed that in experiments using the endothelium specific CD73 mutant, the corresponding loxP controls showed a stronger tumor growth and edema formation when compared to WT controls of the global CD73 knockout. Like other groups [14, 15], we have observed similar effects in chimeric mice in which WT mice with WT bone marrow transplantation showed an attenuated growth of tumor size and edema versus WT mice without transplantation. That genetic background and positional effects may influence the phenotype is well known in the literature [32].

Differences in intestinal commensal microbes, known to be important immune modulators [33] must also be considered. It is becoming increasingly apparent that differences in the gut microbiome can significantly influence the disease expression in rodents [34] and also humans [35]. Routine microbiological monitoring according to FELASA recommendations [36] of the animals used in the present study did not reveal any evidence for common murine pathogens except for Pasteurellaceae and Protozoa. No information is available, however, on the health status of mice from two of the published melanoma studies [16, 17] while Koszalka et al. reported to have used specific pathogen free (SPF) animals [13].

It has been suggested that adenosine may regulate the vascular supply to neoplastic tissue thereby influencing the growth of tumors [37]. More recently we have shown that CD73 in addition influences arteriogenesis [38]. Moreover, CD73 may enhance vascular permeability by induction of adhesion proteins on endothelial cells [39] and the extracellular fluid of solid carcinomas was shown to contain biologically active concentrations of adenosine [40]. Our results are consistent with a role of adenosine in vascular permeability of vessels supplying the tumor in which after intradermal cell application the peritumoral edema was significantly reduced in CD73 mutants. This difference was fully abrogated in chimeric mice with CD73^−/−^ bone marrow suggesting that the CD73 on non-hematopoietic cells (likely endothelial cells) accounts for the reduced peritumoral edema in the global CD73 knockout.

Tumor cells greatly differ in their expression of CD73 and its role on disease progression is somewhat controversial [5, 28]. In a study on human breast cancer cells a positive correlation between CD73 and metastasis was reported [18] while in contrast a later study in breast carcinoma found CD73 to be a marker of good prognosis [19]. Overexpression of CD73 on B16-F10 melanoma cells which were injected intravenously in the murine model showed enhanced metastasis into the lung while similar to our results, local growth was not affected after subcutaneous inoculation [10]. This is in accordance with reports of an association of tumor CD73 with other metastasis promoting antigens and preference for hematogenic metastasis in melanoma [41, 42]. On the other hand, loss of host CD73 did not affect lung metastasis in our experiments in contrast to another report [14].

The B16-F10 melanoma cells used in the present study showed a rather low CD73 activity which matches FACS data from other groups which failed to detect CD73 on the surface of B16-F10 cells [14, 16]. Thus, CD73 on B16-F10 cells can be expected to produce adenosine only at a negligible rate. It is therefore conceivable that the low CD73 expression prevented the generation of an adenosine-mediated immunosuppressive environment involving the activation, clonal expansion and homing of tumor-specific CD4^+^ T helper and CD8^+^ cytotoxic T cells. In this context it is also unknown whether B16-F10 melanoma cells in vivo release similar high quantities of ATP as it was elegantly shown for OVCAR-3 human ovarian carcinoma and MZ2-MEL human melanoma cell lines [24].

The adenosine concentration in the tumor microenvironment depends on the ATP release from tumor cells themselves [2] but also from the invading immune cells [3]. Furthermore, it is the activity of the ATP degrading enzymes CD39 and CD73 which dictates half-life of ATP and rate of adenosine formation. The situation is further complicated by the fact that not only adenosine influences tumor growth but also extracellular ATP acting on P2 receptors [43, 44]. Furthermore, adenosine has been reported to inhibit tumor growth via A3 receptor both directly on melanoma cells [25] and indirectly by activating NK and CD8^+^ cells [9]. It should also be noted, that CD73, aside of its enzymatic activity in producing adenosine, has been reported to bind to the extracellular matrix protein tenascin C which promotes tumor cell migration in a non-enzymatic fashion [11]. Interestingly, binding of CD73 to tenascin C strongly inhibits enzymatic activity of CD73, so that the migration stimulating effect of tenascin C may not be dependent on adenosine [11].

## Conclusions

The present study demonstrates that lack of CD73 on immune and endothelial cells did not cause robust inhibition of tumor growth and metastasis when B16-F10 melanoma cells were applied by different modes. B16-F10 cells showed negligible CD73 activity which limited its contribution to an adenosinergic tumor microenvironment. Under this condition lack of CD73 on host cells alone failed to generate a proinflammatory state which would be of sufficient magnitude to inhibit local tumor growth and metastatic spread. Whether overexpression of ecto-nucleotidases (CD39, CD73) on tumor cells plays a more profound role in adenosine-triggered tumor immune escape cannot not be decided on the results obtained in this study. It is also possible that the relative contribution of tumor versus host CD73 may be different for different tumors.

## Electronic supplementary material

Additional file 1: Figure S1: Immunohistochemistry of carotid artery in Tie2-Cre^+^ CD73 ^flox/flox^ (eCD73^−/−^) mice. Immunohistochemical analysis of carotid artery showed strong CD73 expression (red) on endothelial cells of CD73^flox/flox^ (loxP) mice (A) und lack of CD73 activity on endothelium in eCD73^−/−^ mice (B). Smooth muscle cells were identified by staining with α-SMA (green) and nucleus with DAPI staining (blue). Bars: 200 μm. (DOCX 113 KB)
