# Supplementary material for: Growth and metastasis of B16-F10 melanoma cells is not critically dependent on host CD73 expression in mice
Source: BMC Cancer. 2014 Dec 2;14:898. doi: 10.1186/1471-2407-14-898 (PMC4265456; doi:10.1186/1471-2407-14-898)
Supplement: Supplementary file 1 — Additional file 1: Figure S1: Immunohistochemistry of carotid artery in Tie2-Cre+ CD73 flox/flox (eCD73−/−) mice. Immunohistochemical analysis of carotid artery showed strong CD73 expression (red) on endothelial cells of CD73flox/flox (loxP) mice (A) und lack of CD73 activity on endothelium in eCD73−/− mice (B). Smooth muscle cells were identified by staining with α-SMA (green) and nucleus with DAPI staining (blue). Bars: 200 μm. (DOCX 113 KB) [file 12885_2014_5066_MOESM1_ESM.docx]

|  | **loxP** | **eCD73^-/-^** |
| --- | --- | --- |
| **CD73 / α-SMA / DAPI** | **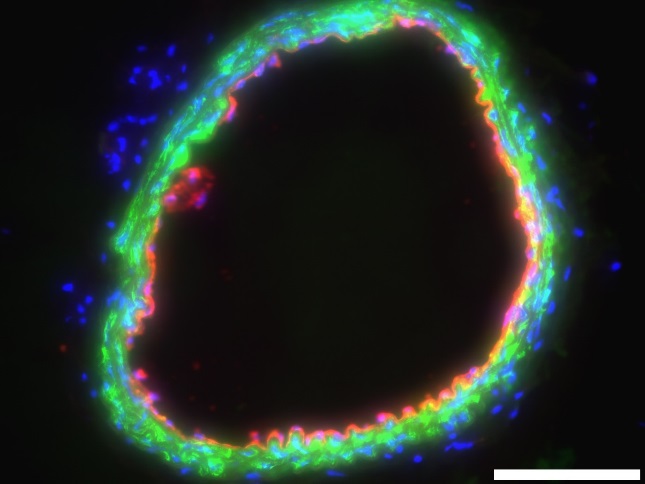**  **A** | **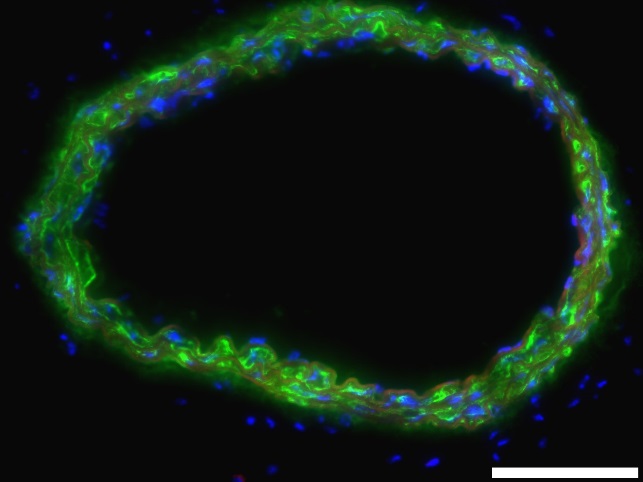**  **B** |

**Additional file 1 Figure S1. Immunohistochemistry of carotid artery in Tie2-Cre^+^ CD73 ^flox/flox^ (eCD73^-/-^) mice.** Immunohistochemical analysis of carotid artery showed strong CD73 expression (red) on endothelial cells of CD73^flox/flox^ (loxP) mice (A) und lack of CD73 activity on endothelium in eCD73^-/-^ mice (B). Smooth muscle cells were identified by staining with α-SMA (green) and nucleus with DAPI staining (blue). Bars: 200 µm.
